# 

**Published:** 1889-12

**Authors:** 


VOL. XIX. DECEMBER, 1889. NO. 12

### Southern Medical Record.

LIBRARY Indexed S. G. O.

EDITORS:

*T. S. POWELL. M. D.* *R. C. WORD. M. D.*

COLLABORATORS:

J. McF. Gaston, M. D.,
Julian J. Chisolm, M. D.,
W. P. Nicolson, M. D.,
E. Van Goidtsnoven, M. D.,
P. R. Cortelyou, M. D.,
Lindsay Johnson, M. D.,
G. G. Roy. M. D.,
A. G. Hobbs, M. D.,
W. S. Elkin. M. D.,
W. A Crow M. D.,
Jno Thad Johnson, M. D.,
K. P. Moore. M. D.

**Terms:—$2.00 per annum, in advance; four copies $6.00; single copies 20 cents.**

**Address R. C. Word, M. D. Managing Editor, Atlanta, Ga.**

Published and entered as Second Class Matter at Atlanta, Ga.

